# Investigation into SiO_2_ Etching Characteristics Using Fluorocarbon Capacitively Coupled Plasmas: Etching with Radical/Ion Flux-Controlled

**DOI:** 10.3390/nano12244457

**Published:** 2022-12-15

**Authors:** Won-nyoung Jeong, Young-seok Lee, Chul-hee Cho, In-ho Seong, Shin-jae You

**Affiliations:** 1 Department of Physics, Chungnam National University, Daejeon 34134, Republic of Korea; 2 Institute of Quantum System (IQS), Chungnam National University, Daejeon 34134, Republic of Korea

**Keywords:** pulsed plasma, SiO_2_ etching, high aspect ratio etching, capacitively coupled plasmas

## Abstract

SiO_2_ etching characteristics were investigated in detail. Patterned SiO_2_ was etched using radio-frequency capacitively coupled plasma with pulse modulation in a mixture of argon and fluorocarbon gases. Through plasma diagnostic techniques, plasma parameters (radical and electron density, self-bias voltage) were also measured. In this work, we identified an etching process window, where the etching depth is a function of the radical flux. Then, pulse-off time was varied in the two extreme cases: the lowest and the highest radical fluxes. It was observed that increasing pulse-off time resulted in an enhanced etching depth and the reduced etching depth respectively. This opposing trend was attributed to increasing neutral to ion flux ratio by extending pulse-off time within different etching regimes.

## 1. Introduction

Stacking up and shrinking down strategies complicate high aspect ratio etching. Etching profile distortions, such as twisting, bowing, necking, etc., are well-known difficulties in high aspect ratio etching processes, which become more severe with increasing aspect ratio [1,2,3,4]. To tackle such problems and achieve ideal etching profiles, pulse-modulated discharges have been widely adopted in industry due to their technical benefits. Without changing any of the equipment, one can obtain additional control parameters, namely pulse-frequency and duty ratio for each power generator. In other words, compared to conventional continuous discharges, pulse-modulated discharges have at least two more knobs that can be tuned for process optimizations. Introducing control parameters by adopting pulse modulation has provided the possibility of exploring a wide range of process windows that are free from distortions. Indeed, it has been shown that pulse-modulated plasma discharges ameliorate the undesirable phenomena, mainly due to the reduction of local charge build-up inside of the features [5,6,7].

Together with the profile distortions, low productivity, i.e., a low etching rate, is another big challenge in high aspect ratio etching processes. Etching rate reduction as the aspect ratio increases is known as aspect ratio dependent etching (ARDE). The causes of such phenomenon are well reviewed [8]. Currently, the dominant cause is considered as a transport-related issue [9,10,11]. Huard et al. investigated ARDE through etching profile and plasma simulations, in which a wide range of parameters that are strongly related to transport phenomena, such as ion angular distribution, surface recombination rate, etc., were varied to scale ARDE [12]. Huang et al. studied detailed kinetics of ions and neutrals under various conditions via etching profile and plasma simulations [13]. Because we cannot directly observe ion and neutral transport inside the high aspect ratio features, numerical studies are appropriate to investigate detailed kinetics. Verification by means of experiments should be conducted with internal parameters controlled. To confirm internal parameters, which dominate the etching results, plasma diagnostics are necessary. However, there are few works comprising etching experiments in tandem with internal parameter control based on plasma diagnostics.

In this work, we investigated plasma etching characteristics under discharge conditions through cross-section morphology measurement of etched samples as well as plasma parameters measured through various techniques. First, the fluorocarbon to argon gas ratios were varied with fixed pressure, total flow rate, and RF power. Then, pulse-off time was varied to control the ratio of radical to ion flux to the wafer in two extreme cases: the lowest and highest radical fluxes [14,15]. Through plasma diagnostics, it was observed that the radical fluxes in continuous wave discharges and radical to ion flux ratios in pulse-modulated discharges were individually varied. Based on this, the etching characteristics under the conditions are discussed in this paper.

## 2. Materials and Methods

**Figure 1:**
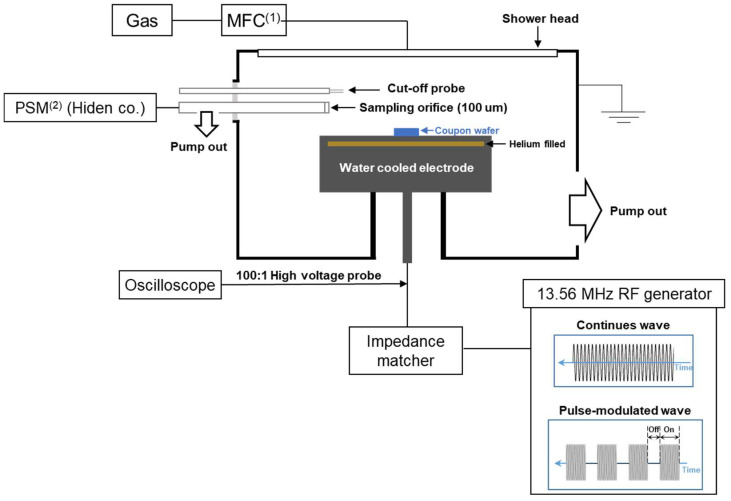
Experimental setup. (1) Mass flow controller, (2) Plasma sampling mass spectroscopy.

Figure 1 shows a schematic of the experimental setup. The plasma chamber has a 40 cm diameter and a 12 cm gap distance from the electrode to the counter electrode. Chamber wall is grounded. The chamber is evacuated with a dry pump and turbo molecular pump combination at a base pressure of ~10^−5^ Torr. Feed stock gases are fed by a mass flow controller to the chamber through a shower head. A mixture of argon and C_4_F_8_ was used in this experiment. To sustain the plasma discharge, 13.56 MHz radio-frequency generators are used with an impedance matching network. The RF generators are equipped with a pulse-modulation unit. The electrode temperature was maintained at 10 °C by an individual chiller with a helium backside cooling system.

In the etching experiment, coupon wafers were used. Each sample is composed of a patterned amorphous carbon (ACL) mask on a 2.4-µm-thick silicon dioxide layer deposited by plasma enhanced chemical vapor deposition on a silicon substrate. Trench patterns have various width from 60 nm to 200 nm. A mass spectrometer, PSM (Hiden. Co., Warrington, UK) is attached to the chamber with a 100-µm sampling orifice, as shown in Figure 1. The mass spectrometer is evacuated with a differential pumping unit at a base pressure of ~10^−7^ Torr. A cut-off probe was inserted into the chamber directly above the sampling orifice. The spacing between two antennas of the cut-off probe is 1 cm. Due to the limited access to the electrode during the discharge, a high voltage probe was attached near the electrode. To analyze pulse-modulated etching experiments in detail, the following plasma parameters were measured: radical densities, electron densities, and self-bias voltages.

In this work, we used refined appearance potential mass spectrometry to measure the absolute radical densities, which is well described elsewhere [16]. Here, we briefly explain the main idea of such method. A typical signal of a neutral species X detected by a quadrupole mass spectrometer is given by

(1) $$S_{X}\left(E\right)=\alpha I_{e}n_{X}\sigma_{X\to X^{+}}\left(E\right).\;$$

where *E* is electron energy colliding with a neutral gas particle *X*, *S* is the signal, *α* is a coefficient related to the measurement geometries and the neutral particle mass to charge ratio, $I_{e}$ is the electron current in the ionizer, *n_x_* is the species density at the ionizer, and *σ* is the ionization cross-section of the species. Based on Equation (1), the neutral species density can be obtained by

(2) $$n_{X}=\left(\frac{S_{X}\left(E\right)}{S_{Ar}\left(E\right)}\right)\left(\frac{m_{X}}{m_{Ar}}\right)\left(\frac{\sigma_{Ar\to Ar^{+}}\left(E\right)}{\sigma_{X\to X^{+}}\left(E\right)}\right)n_{Ar}\;.\;$$

According to the definition of *α*, the *α* coefficient ratio between two different species in the identical measurement setup is equal to the mass ratio because those species have the same electrical charge by direct ionization process in the measurement system. To evaluate the $n_{x}$, a reference argon signal, $S_{Ar}\left(E\right)$, was obtained from pure argon gas in the chamber at a pressure of 20 mTorr, and cross sections were taken from the Korea Institute of Fusion Energy, Data Center for Plasma Properties.

The electron densities were measured by cut-off probe diagnostics using a wave cut-off phenomena in plasmas, through a network analyzer [17,18,19,20]. From the measured cutoff frequencies, we can directly estimate the electron density by using the equation below

(3) $$f_{cutoff}=8980\sqrt{n_{e}}\;.\;$$

where the electron density,$n_{e}$, is in units of cm^−3^ and the cut-off frequency is in units of Hz. Cut-off probe and sampling orifice are not located in the center of the chamber. All experiments in this work were carried out in 20 mTorr pressure. In a low pressure regime, a typical density profile is well known [21]. Thus, spatial variation of charged or neutral particle densities from the center to measurement location are nearly negligible.

The self-bias voltages were obtained from a high-voltage probe near the electrode by averaging the voltage waveform over the RF period.

Based on the plasma diagnostic techniques described above, we investigated the plasma discharge window where the radical and ion fluxes can be controlled. To obtain such a window, fluorocarbon to noble gas ratio and pulse-off time were adjusted. Throughout the investigation, total flow rate 100 sccm, 300 W power, and 20 mTorr pressure were sustained. In pulse modulated conditions, pulse-on time was fixed at 1 ms, while the pulse-off time was varied from 0.1 to 1 ms. An illustration of a typical waveform used in this work is shown in Figure 1.

## 3. Results and Discussion

### 3.1. Fluorocarbon to Argon Gas Ratio Variations

**Figure 2:**
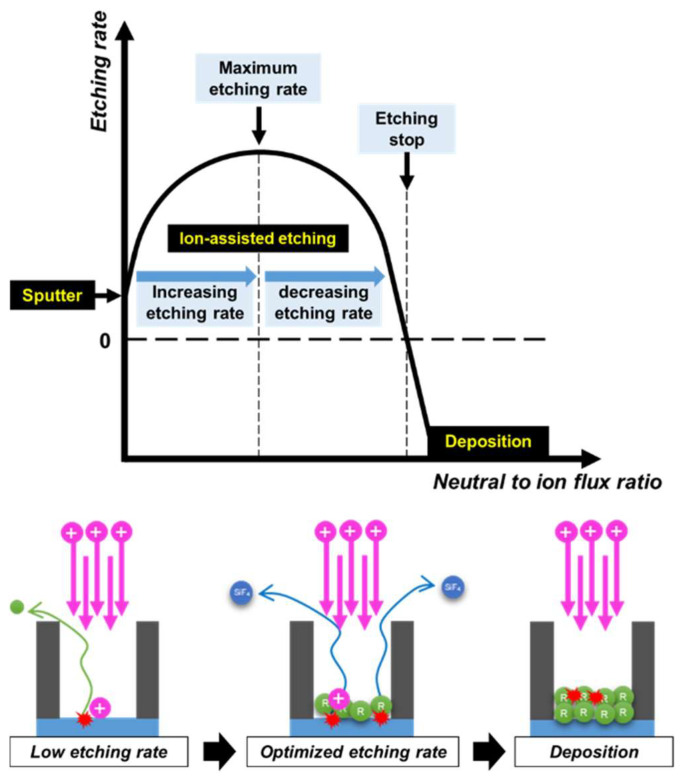
Illustration of etching regimes regarding different neutral to ion flux ratios with fixed ion energy.

In an oxide etching process using fluorocarbon plasma, a target material is subjected to energetic ion and radical fluxes simultaneously. The former provides energies, being used for breaking Si-O bonds as well as chemical reactions, and the latter supplies chemical reactants. Depending on the ratio of neutral and ion fluxes with the ion energy, a variety of etching behaviors exist. Previous studies investigated etching regime transitions with different ion energies [22,23]. Here, based on these previous studies, it can be deduced that the three etching regimes could also be characterized with variations in neutral flux with a fixed ion flux and energy, as shown in Figure 2. Although the idea has already been stated [8], experimental validation has not been made. The two extreme regimes, sputtering and deposition, are characterized by negligible etching rates, respectively, due to the depletion of chemical reactants and excessive polymer deposition. Ion-assisted etching takes place between the two extreme regimes, where an increasing neutral flux initially elevates the etching rate until reaching a maximum, after which the etching rate is continuously reduced, eventually entering the deposition regime. 

**Figure 3:**
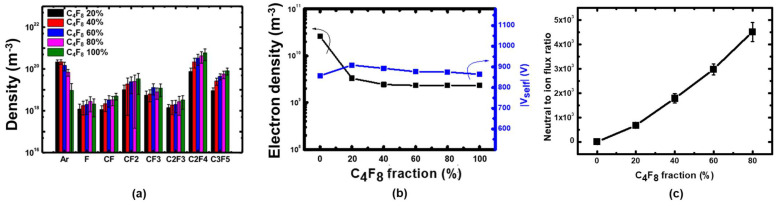
(**a**) Absolute neural species densities, (b) electron densities and self-bias voltages in the continuous wave discharges with various C_4_F_8_ fractions, (**c**) calculated neutral to ion flux ratios based on the measurement data.

In the first experiment to investigate etching characteristics under various neutral fluxes, first we carried out plasma diagnostics to figure out the plasma window where radical fluxes can be individually controlled. Second, we conducted etching experiments within the window. Figure 3a presents the absolute radical densities measured through the PSM at a pressure of 20 mTorr with the gas mixture variations. Under a fixed total flow rate (100 sccm), fractional C_4_F_8_ were varied from 20% to 100% with a 20% interval. In the same condition, Figure 3b shows measured electron densities and self-bias voltages. Compared to pure argon plasma (C_4_F_8_ 0%), introducing 20% C_4_F_8_ gas reduced electron density, but increased self-bias voltage. The electron density reduction is mainly because of negative ions created by electron attachment. The reduced electron density increases the sheath voltage. A further increase of C_4_F_8_ fraction did not significantly affect electron densities or self-bias voltages. On the other hand, as observed in Figure 3a, the CF*_x_* and C*_x_*F*_y_* species densities increase with increasing C_4_F_8_ gas fraction, i.e., C_4_F_8_ partial pressure.

Radicals are electrically neutral, and their flux into the surface is described by

(4) $$\Gamma_{r}=\frac{1}{4}n_{r}\sqrt{\frac{8kT_{r}}{\pi m_{r}}}\;,$$

where $\Gamma_{r}$ is the flux of the species to the surface, $n_{r}$ is the density of the species, k and $T_{r}$ are the Boltzmann constant and temperature respectively, and $m_{r}$ is the mass of the species. Each species presented in Figure 3a may have different contributions to the silicon oxide etching process. Consideration of such aspects requires information on the chemical reactivities of each species, for example. However, the sticking coefficients of each species are not well-known for surfaces: ACL, silicon oxide, as well as polymer. Furthermore, the transport process of radicals and ions through the trench structures may greatly alter the radical and ion fluxes reaching the etch front depending on the structure geometries and sticking coefficients. In this work, for the purpose of simplicity, we considered integrated radical fluxes at the wafer surface excluding stable species: Ar and C2F4. And temperatures of each species were assumed to be room temperature (300 K). Additionally, decaying characteristics of radical species in pulse-off time can greatly alter the radical composition. In this work, the measured radical densities in pulsed plasma can be treated as time-averaged quantities, which include information of such characteristics. The specific mechanism of each radical species may elucidate important aspects. In this work, however, we did not investigate detailed kinetics of each radical species in pulse-off time.

In typical low-pressure plasmas, electrons have much higher temperatures (3–5 eV) than ions and radicals due to the poor energy transfer efficiency from electrons to heavy particles. Accordingly, ions and radicals are nearly room temperature (0.026 eV), and thus the radical densities and radical fluxes are linearly dependent. Ion fluxes are described by the Bohm flux, which is the ion species density at the sheath edge times Bohm velocity

(5) $$\Gamma_{i}=n_{is}u_{B}\;.$$

where $\Gamma_{i}$ is the ion flux, $n_{is}$ is the ion density at the sheath edge, and $u_{B}$ is the Bohm velocity. Ion compositions were not measured in this work, ion fluxes were calculated with measured electron density with estimated Bohm velocity; electron temperature was assumed to be 3 eV and argon ion mass was used in the calculation. In an electronegative plasma, negative ions are trapped in the bulk plasma, creating an electronegative core because they do not have enough energy to overcome the presheath potential. The quasi-neutral condition at the sheath edge is satisfied by ions and electrons only [21]. Thus, measured bulk electron densities, though not the exact center of the discharge, are equal to the ion density at the sheath edge. The Bohm velocity depends on the square root of the electron temperature. Considering a typical electron temperature in a low-pressure plasma (3–5 eV), the Bohm velocity does not significantly affect the ion flux compared to the density. Similar to the radical fluxes, ion fluxes to the surface also linearly depend on the ion densities. Ion energies at the electrode can be represented by self-bias voltages. From the above observations, we conclude that the radical fluxes are rather controllable parameters by changing the C_4_F_8_ to argon gas ratios, compared to the ion fluxes and ion energies. Resulting neutral to ion flux ratios are presented in Figure 3c.

**Figure 4:**
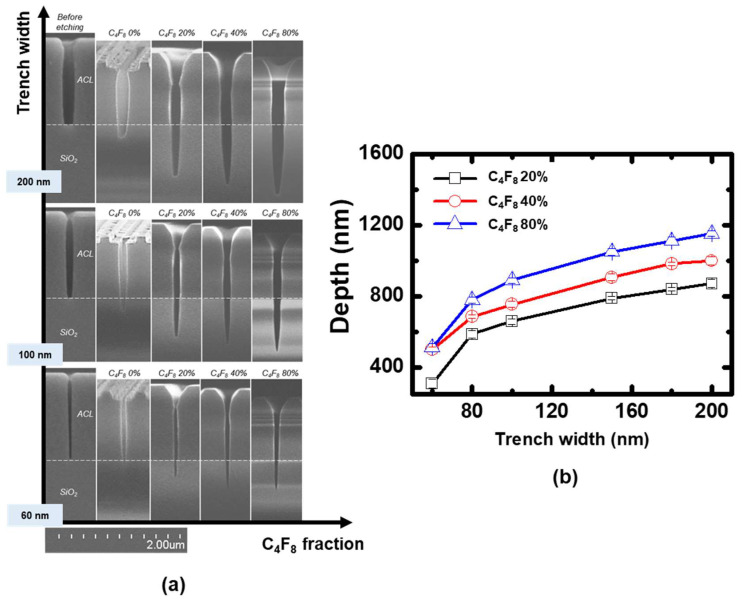
(**a**) Etching profiles measured through SEM, (**b**) measured silicon oxide etching depth under the continuous wave discharges with various C_4_F_8_ to argon gas ratios.

Coupon wafers were etched under the same conditions as above, and their etching profiles measured through SEM are shown in Figure 4. During the etching of coupon wafer under the C_4_F_8_ 0% condition, where only energetic ions contribute to the etching process, sputtering of silicon oxide by energetic ion bombardment is the only way of removing the oxide. When 20% C_4_F_8_ is introduced, the etching depth of the oxide greatly increased compared to the 0% condition. This trend is observed from all trench widths we investigated, as shown in Figure 4. As C_4_F_8_ gas ratio further increased to 40% and 80%, oxide etching depths increased for all trench widths.

The etching characteristics of silicon oxide under the C_4_F_8_ to argon gas ratio variations can be concluded as follows. As the C_4_F_8_ gas fraction increased, the electron density of the bulk plasma and mean ion energy were barely altered as shown in Figure 3, while the neutral fluxes significantly increased in comparison. Considering the plasma characteristics, coupon wafers were subjected to nearly constant ion fluxes and energies with varying neutral fluxes under the present conditions. Thus, the etching depth enhancement by increasing the C_4_F_8_ gas fraction is attributed to the increased neutral fluxes. Furthermore, from the ion-neutral synergetic model as shown in Figure 2, the results are located in the early stage of ion-assisted etching regime considering the etching depth dependence on the neutral fluxes. The neutral flux and the etching depth are monotonically dependent due to the limited neutrals, or the so-called neutral-rare condition. In other words, though the etching depth was increased with the increase of neutral fluxes, the etching regime is still located in the initial stage of ion-assisted etching, not yet reaching the maximum etching depth.

### 3.2. Effects of Extended Pulse-Off Time in the Neutral-Rare Condition

**Figure 5:**
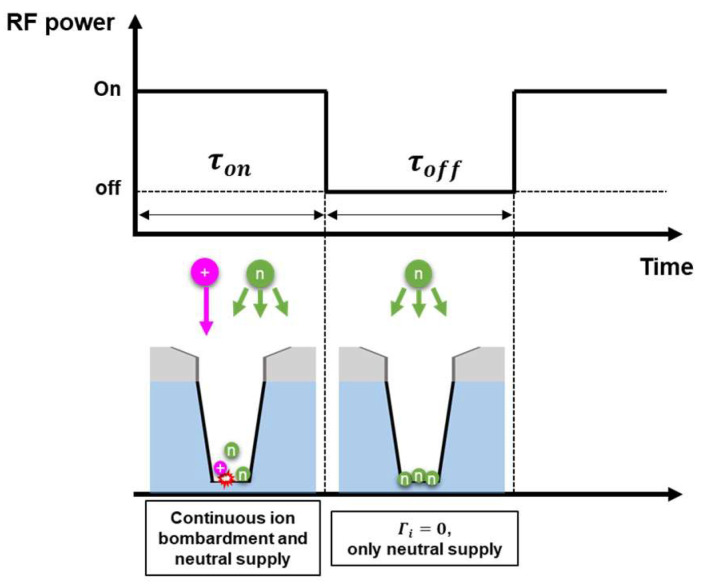
A simple pulsed plasma model.

In an etching process using continuous wave plasmas, the target material is subjected to continuous ion and neutral fluxes. In a pulsed plasma, on the other hand, the ion and neutral fluxes incident to the target material have a time dependence. Ion fluxes are strongly dependent on the pulse modulation, while neutral fluxes are nearly constant over a pulse period [24,25]. A simple model accounting for pulse modulation effects on the ion and neutral fluxes is shown in Figure 5. To confirm the pulse-off time effects, we carried out etching experiments using pulse-modulated plasmas with a varying pulse-off time and a fixed pulse-on time.

**Figure 6:**
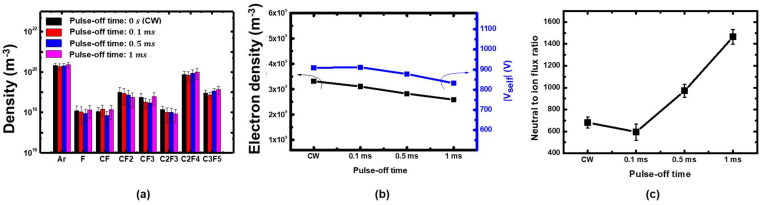
(**a**) Absolute neural species densities, (**b**) electron densities and self-bias voltages, (**c**) calculated neutral to ion flux ratios based on the measurement data in the pulse-modulated plasmas with various pulse-off time in C_4_F_8_ 20%.

Figure 6a exhibits the absolute radical densities measured through the PSM at a pressure of 20 mTorr with pulse-off time variations. The C_4_F_8_ gas fraction was maintained at 20%. It is observed that the CF*_x_* and C*_x_*F*_y_* radical species densities were not significantly varied under the pulse-off time variations. Figure 6b shows the electron densities and self-bias voltages measured in the same conditions. Increasing pulse-off time barely affected the electron densities and self-bias voltage. From the above observations, the radical and electron densities, as well as the self-bias voltages, we expect that the radical and ion fluxes incident to the coupon wafer are almost constant in the pulse-on period regardless of pulse-off time. 

In this system, based on the simple model shown in Figure 5, one of the important factors determining the neutral to ion flux ratio is pulse-off time. The coupon wafer is subjected to constant neutral flux for the whole period, while it takes constant ion fluxes only in the pulse-on period. Thus, extending the pulse-off time in a fixed pulse-on time leads to an increasing neutral to ion flux ratio, resulting in an etching depth elevation or degradation depending on the etching regime as described in Figure 2. Calculated neutral to ion flux ratios based on the simple pulsed plasma model are shown in Figure 6c. Although the neutral to ion flux ratio slightly decreased in the 0.1 ms pulse-off time condition compared to the continuous wave discharge, it shows an increasing trend with extending pulse-off time.

**Figure 7:**
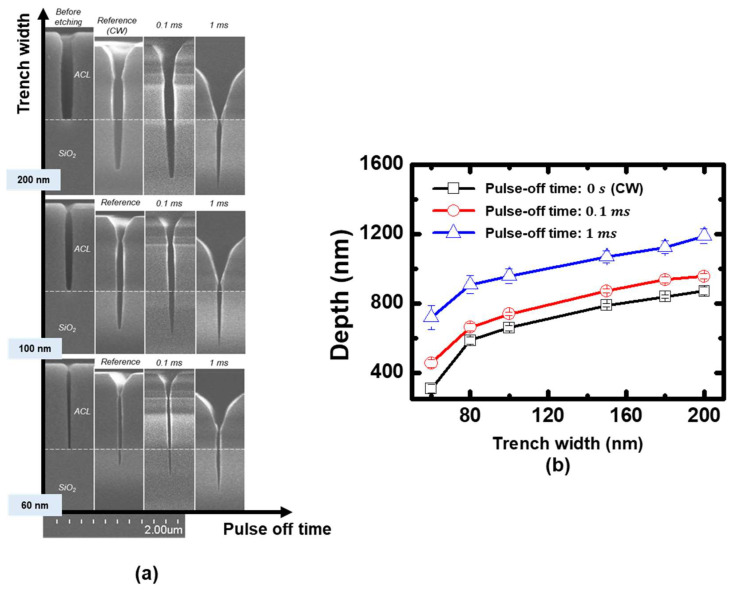
(**a**) Etching profiles measured through SEM, (**b**) measured silicon oxide etching depth under the pulse-modulated plasmas with various pulse-off time in C_4_F_8_ 20%.

Coupon wafers were etched using the pulsed plasmas under the same conditions as Figure 6, and their measured etching profiles are shown in Figure 7. It is observed that increasing pulse-off time resulted in increasing etching depths at all trench widths. This can be attributed to the increased neutral to ion flux ratio similar to the results shown in Figure 4. The etching profile under the continuous wave condition in Figure 7 was identified to be in the initial stage of the ion-assisted etching regime, before the maximum etching depth. Here, increasing the neutral to ion flux ratio by extending the pulse-off time resulted in the enhanced etching depth shown in Figure 7. 

### 3.3. Effects of Enhanced Neutral to Ion Flux Ratio in the Neutral-Rich Condition

**Figure 8:**
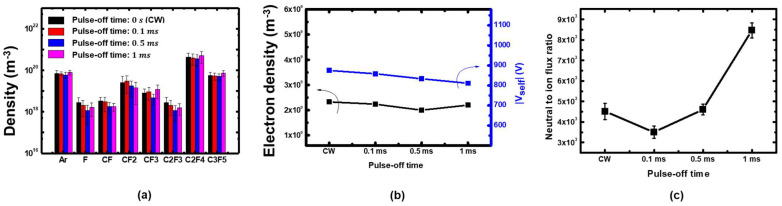
(**a**) Absolute neural species densities, (**b**) electron densities and self-bias voltages, (**c**) calculated neutral to ion flux ratios based on the measurement data in the pulse-modulated plasmas with various pulse-off time in C_4_F_8_ 80%.

We also applied the extended pulse-off time to the case where the C_4_F_8_ fraction is 80%, which is believed to be in the neutral-rich condition. Radical and electron densities and self-bias voltages measured in the window were nearly constant within a few percent under the pulse-off time variations, as shown in Figure 8. Thus, analogous to the previous section, it is expected that the extending pulse-off time also increases the neutral to ion flux ratio in this condition. Calculated neutral to ion flux ratios based on the simple pulsed plasma model is presented in Figure 8c. Although the neutral to ion flux ratio slightly decreased in the 0.1 ms pulse-off time condition compared to the continuous wave discharge, it exhibits an increasing trend with extending pulse-off time.

**Figure 9:**
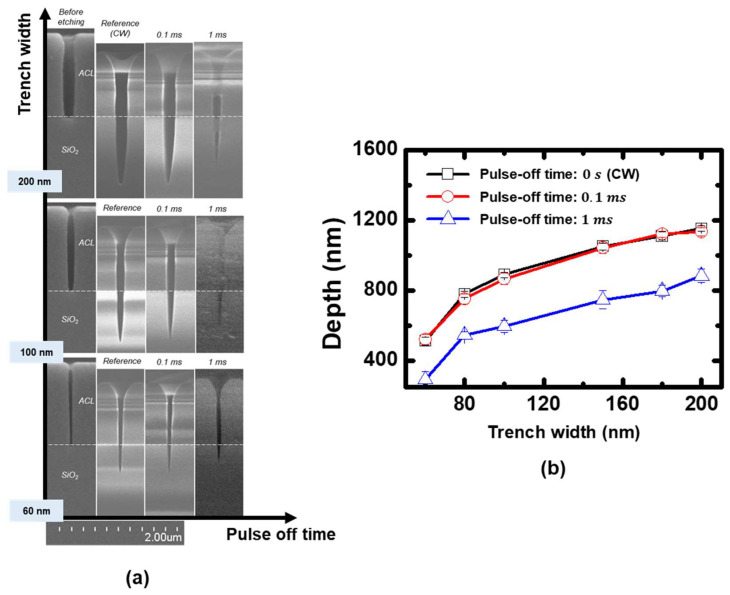
(**a**) Etching profiles measured through SEM, (**b**) measured silicon oxide etching depth under the pulse-modulated plasmas with various pulse-off time in C_4_F_8_ 80%.

Figure 9 shows etching profiles with increasing pulse-off time in 80% C_4_F_8_. Contrary to the previous results in which the etching depth trend increased with pulse-off time variations in 20% C_4_F_8_, in this case the etching depths decreased with increasing pulse-off time. This opposing trend can be interpreted as follows. It was shown that the radical fluxes can be nearly individually controlled by adjusting the C_4_F_8_ to argon gas ratio. Moreover, increasing neutral fluxes by setting the C_4_F_8_ gas fraction from 20% to 80% yielded increasing etching depth, as shown in Figure 4. Here, extending the pulse-off time in the C_4_F_8_ 80% condition is the equivalent to further increasing the neutral flux to the coupon wafer. Based on the model described in Figure 2, the opposing trend is attributed to excessive neutral flux after reaching the maximum etching depth, leading to the etching depth reduction. 

## 4. Conclusions

In this work, the etching characteristics of silicon oxide in a wide range of radical to ion flux ratios were investigated. Although the etching profiles presented in this work do not exactly match with those desired in actual device fabrication, considering the focus of this work, which was to investigate etching behavior depending on the plasma parameters in high aspect ratio features, our results can be thought of as meaningful. Adjusting the fluorocarbon to noble gas ratio and pulse-off time was shown to successfully control the radical flux and radical to ion flux ratio, respectively. We identified the etching process window, where the etching depth is monotonically dependent on the radical flux, the early stage of the ion-assisted etching regime. Extending the pulse-off time in the lowest fluorocarbon gas ratio condition, in which the radical fluxes were shown to be rare, resulted in an increase of the etching depth. A decreasing etching depth, on the contrary, was observed by extending the pulse-off time in the highest fluorocarbon gas ratio condition. We experimentally confirmed the silicon oxide etching rate trend by increasing the radical to ion flux ratios, and the results exhibited good agreement with the simple model described in this paper. The present results can be applied to optimizing radical to ion flux ratios in various conditions. 

## Data Availability

Data presented in this article is available on request from the corresponding author.
